# Remarks on Myeloid Sarcoma in Children

**DOI:** 10.4274/tjh.galenos.2019.2019.0002

**Published:** 2019-05-03

**Authors:** Sevgi Gözdaşoğlu

**Affiliations:** 1 Retired Professor of Pediatrics, Hematology and Oncology

**Keywords:** Myeloid sarcoma, Cytogenetic, Prognosis

## To the Editor,

Arslantaş et al. [1] reported “A Rare Cause of Paraplegia: Myeloid Sarcoma” in a recent issue of this journal. I would like to remark on a few points not mentioned in that paper. 

Extramedullary infiltrations (EIs) of the soft tissue, also known as myelosarcoma (MS) or granulocytic sarcoma (GS), occur in approximately 4% to 5% of children with acute myeloid leukemia (AML) in western countries [2]. MS may develop before, during, or after the occurrence of AML. AML is a clinically and genetically heterogeneous disease. Immunohistochemistry and immunophenotyping are important for the accurate diagnosis of AML. White blood cell (WBC) count at diagnosis, FAB subtypes, and cytogenetics are the main important prognostic factors. For that reason, these analyses should be performed for all patients [3,4,5]. 

Cytogenetic analysis has become an important parameter for the diagnosis, prognosis, and treatment selection of AML. t(8;21) is the most common abnormality and it is primarily found in the M2 subtype. The inv(16) and t(16;16) associated with M4Eo and t(15;17) and t(11;17) associated with the M3 subtype are favorable, whereas 11q23 associated with M4 and M5 variants is found unfavorable for prognosis [2]. Xu et al. [5] reported that monosomal karyotypes are independent risk factors for poor prognosis. 

The prognostic significance of MS in childhood AML is still controversial. Some groups reported an unfavorable prognosis but others demonstrated a favorable outcome [3,4,6]. Central nervous system leukemia and MS together with high initial WBC count at diagnosis are high risk factors for relapse [6].

**Figure 1:**
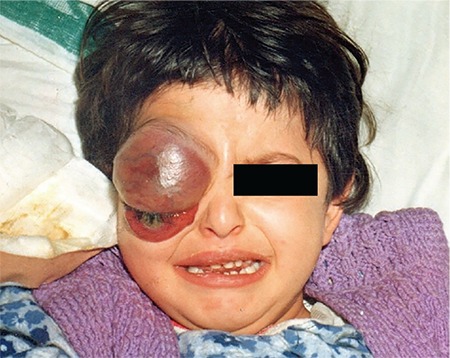
Appearance of exophthalmos, proptosis, chemosis, and orbital mass.

Orbital granulocytic sarcoma (OGS) was first reported in 1971 by Çavdar et al. [7] from Turkey. Some researchers in Turkey also reported that there was a connection between AML and EI in several retrospective analyses of patients as well as in some case reports [8,9,10]. Çavdar et al. [7] analyzed 33 patients presenting with OGS characterized by exophthalmos, proptosis, chemosis, and orbital masses (Figure 1). OGS was noted in 33 (27%) of 121 patients. These patients were compared with 41 cases of AML without OGS seen during the same period. The majority of the patients with OGS were of low socioeconomic status. The mean age was 6.7 years and 24 of the patients were male while 9 were female. OGS occurs in patients with M4 or M5 subtypes. Hematological findings in the two groups were not significantly different. Cytogenetic study revealed that the t(8;21) abnormality was frequent. The expression of tissue adhesion molecules CD56 and CD44 and the expression of MDR (p-gp) were more common in OGS cases. These findings might explain the different prognosis in patients with OGS [4,11]. The mean survival time of 8.7 months in the OGS group was significantly shorter than that of patients without OGS (28.6 months) (p<0.01) treated before 1990 [4]. Çavdar et al. [11] suggested that this type of presentation could indicate a special high-risk biological entity. Further molecular and therapeutic studies are required for a better understanding of the reasons for tissue involvement and to choose the most effective treatment options.
