# Supplementary material for: Remarks on Myeloid Sarcoma in Children
Source: Turk J Haematol. 2019 May 3;36(2):122–3. doi: 10.4274/tjh.galenos.2019.2019.0002 (PMC6516101; doi:10.4274/tjh.galenos.2019.2019.0002)
Supplement: Related texts

# Reply to the Authors

**Authors:** Esra Arslantaş, Cengiz Bayram, Işık Odaman Al, Ezgi Uysalol, Ayça İribaş, Hilal Akı, İbrahim Adaletli, Ali Ayçiçek, Nihal Özdemir

## To the Editor,

We read the letter regarding our publication, “A Rare Cause of Paraplegia: Myeloid Sarcoma”.

Because of its different localization and symptoms, myeloid sarcoma is difficult to diagnose, in particular in patients without initial bone marrow involvement. Thus, the correct diagnosis of MS is often delayed and the misdiagnosis rate is high. Our patient was referred to us with a complaint of hemiparesis and a subsequent thoracolumbar mass detected by magnetic resonance imaging, and no blasts were detected on peripheral blood film but bone marrow aspiration showed blasts compatible with AML. In this regard, the aim of our publication was to highlight to diagnostic challenges and rare presentations of MS in childhood.

We believe, like the author of the preceding letter, that immunohistochemistry, immunophenotyping, and cytogenetic and molecular examinations play an important role in the diagnosis and prognosis, and before creating a treatment plan these should be performed for all patients. Radiology, histology, immunophenotyping, and molecular analyses are all essential for risk stratification and treatment planning. The next step is giving AML-based systemic chemotherapy and, in some cases, as in our case, surgery and/or radiotherapy may be indicated.

We could not mention the clinical, cytogenetic, and molecular features of MS extensively, as our publication was in the format of a Letter to the Editor. We would like to thank the author for her valuable contribution. 

**Best Regards,**
